# Imitation of action-effects increases social affiliation

**DOI:** 10.1007/s00426-020-01378-1

**Published:** 2020-07-14

**Authors:** David Dignath, Gregory Born, Andreas Eder, Sascha Topolinski, Roland Pfister

**Affiliations:** 1 Department of Psychology, University of Freiburg, Engelbergerstrasse 41, 79085 Freiburg, Germany; 2 Department of Psychology, University of Würzburg, Würzburg, Germany; 3 University of Cologne, Cologne, Germany; 4 Division of Neurobiology, Department Biology II, LMU Munich, Munich, Germany

## Abstract

Imitating someone’s actions influences social-affective evaluations and motor performance for the action model and the imitator alike. Both phenomena are explained by the similarity between the sensory and motor representations of the action. Importantly, however, theoretical accounts of action control hold that actions are represented in terms of their sensory effects, which encompass features of the movement but also features of an action’s consequence in the outside world. This suggests that social-affective consequences of imitation should not be limited to situations in which the imitator copies the model’s body movements. Rather, the present study tested whether copying the perceived action-effects of another person without imitating the eventual body movements increases the social-affective evaluation of this person. In three experiments, participants produced visual action-effects while observing videos of models who performed either the same or a different movement and produced either the same or a different action-effect. If instructions framed the action in terms of the movement, participants preferred models with similar movements (Experiment 1). However, if instructions framed the action in terms of the to-be produced action-effect in the environment, participants preferred models with similar action-effects (Experiments 2 and 3). These results extend effect-based accounts of action control like the ideomotor framework and suggest a close link between action control and affective processing in social interactions.

**Electronic supplementary material:**

The online version of this article (10.1007/s00426-020-01378-1) contains supplementary material, which is available to authorized users.

## Introduction

Imitation is often defined as the observation and replication of an action (Romanes 1882) and it serves functions that are crucial for social interaction (Heyes, 2018; Whiten & Ham, 1992). For instance, imitating another person’s action (either simultaneously or with a temporal delay) was proposed to act as a social glue that fosters interpersonal affiliation. Support for this notion comes from studies showing that imitation increases sympathy and prosocial behavior (e.g., Catmur & Heyes, 2013; Chartrand & Bargh, 1999; van Baaren, Holland, Steenaert, & van Knippenberg, 2003). This research most typically analyzed the imitation of perceived movements of another person (i.e., *how* an action was performed by this person). In addition to such perceptions, the action could be also perceived in terms of the goal or desired end-state that was pursued with that action (i.e., *which* action-effect was produced by a specific action). In fact, theoretical accounts of action control hold that actions are represented in terms of their sensory effects, which encompass both features of the movement, but also features of an action’s effect in the world (Hommel, Müsseler, Aschersleben, Prinz, 2001; Powers, 1973). This view suggests that social-affective consequences of imitation should not be limited to situations in which the imitator copies the model’s body movements. The present study provides evidence for this reasoning and shows that the reproduction of action-effects produced by another person has favorable social consequences even when the producing movements are dissimilar.

### Behavior and social consequences of imitation

Imitation can occur spontaneously even without explicit intention to copy somebody else’s actions (Heyes, 2011), e.g., when perceiving the gesture of another person makes people adopt a similar expression (e.g., Chartrand & Bargh, 1999; Colton, Bach, Whalley, & Mitchell, 2018). Furthermore, observation of the movement of another person facilitates execution of a similar action. More specifically, in a study by Brass et al., participants had to perform finger movements according to a visual symbolic cue. Critically, together with the cue, participants were also presented with the video of a finger movement (Brass, Bekkering, Wohlschläger, & Prinz, 2000). Results showed that participants were faster to initiate their finger movement if the perceived action and the to-be performed action matched compared to situations in which the perceived and the to-be performed action differed (see also Catmur, 2016; Pfister, Dignath, Hommel, & Kunde, 2013; for other effector systems than fingers, see Dignath & Eder, 2013; Kilner, Friston, & Frith, 2007; Leighton & Heyes, 2010).

Even more important for the present research, it has been suggested that imitation of the behavior of another person produces not only cognitive, but also social-affective consequences. For instance, studies have shown that imitation increases liking of another person (Catmur & Heyes, 2013; Dignath, Lotze-Hermes, Farmer, & Pfister, 2018), promotes prosocial behaviour (van Baaren, Holland, Steenaert, & van Knippenberg, 2003; van Baaren, Holland, Kawakami, & van Knippenberg, 2004), increases empathy for others (de Coster, Verschuere, Goubert, Tsakiris, & Brass, 2013) and reduces stereotyping (Inzlicht, Gutsell, & Legault, 2012).

### An ideomotor account of imitation

Both motor and social-evaluative effects of imitation have been explained by the similarity of perceived and executed actions (for recent reviews, see Heyes, 2011, regarding the motor effects of imitation; and Hale & Hamilton, 2016, regarding socio-evaluative effects of imitation). But what exactly is a ‘similar’ action? While some theoretical accounts have argued that similarity is the result of a conceptual matching between two events (Jansson et al., 2007), others suggested that similarity is the result of an associative learning process (Heyes, 2001). The present research, however, follows yet another account and adopts an ideomotor perspective on imitation (Prinz, 2005; Brass & Heyes, 2005). According to this view, actions are selected, initiated, and controlled by representations of their sensory consequences, that means, their action-effects (James, 1890; Greenwald, 1970; Hommel, 2013; for a review, Shin, Proctor, & Capaldi, 2010). Specifically, it is assumed that agents learn to associate specific movements with specific sensory effects. Importantly, the associative link between the movement pattern and the action-effect is bidirectional, which implies that the cognitive activation of the action-effect precedes the movement (Dignath, Kiesel, Frings, & Pastötter, 2020; Kühn, Keizer, Rombouts, & Hommel, 2011; van Steenbergen et al., 2017) and that activation of the action-effect re-activates the movement pattern that caused this action-effect (e.g., Dignath, Pfister, Eder, Kiesel, & Kunde 2014 & Hommel, 2001; Kunde, 2003; Pfister & Kunde, 2013). The mental representation of actions in terms of their perceivable effects implies that ‘motor codes’ and ‘perceptual codes’ have a commensurable representational format (Hommel, Müsseler, Aschersleben, & Prinz, 2001). This common coding principle explains a similarity between perceived and performed actions with a code overlap on the representational level (Prinz, 2005).

The ideomotor framework thus provides a straightforward account of imitation because observing another person’s action should automatically activate matching movement patterns in the observer (Prinz, 2005; Wohlschläger, Gattis, & Bekkering, 2003). The stronger the overlap between the codes that represent the perceived event and the intended action, the stronger the tendency to copy the observed action. Furthermore, because both perceived changes of the body and perceived changes in the environment can guide actions (e.g., Pfister, Janczyk, Gressmann, Fournier, & Kunde, 2014a, b; see also Pfister, 2019), imitation is not restricted to the observation of other people’s movements, but also extends to the perception of other people’s action-effects in the environment (Ondobaka, de Lange, Newman-Norlund, Wiemers, & Bekkering, 2012; Wohlschläger & Bekkering, 2002). For instance, Bekkering et al. asked pre-school children to imitate a confederate, who reached with one or both of her hands to one or both of her ears (Bekkering, Wohlschläger, & Gattis, 2000). Movements could be either ipsilateral (e.g., left hand to left ear) or contralateral (e.g., left hand to right ear). Although children were very good at touching the correct ear, they had problems choosing the appropriate hand to perform the movement. This impairment was especially pronounced for trials in which the movement of only one hand had to be imitated. According to the ideomotor view on imitation, children in this study represented their actions in terms of the location of the ear.

### The present research: social consequences of action-effect imitation

The selective review presented above suggests that imitation has motor and social-evaluative consequences that are explained by the ideomotor framework with an overlap between perceptual and motor representations. Furthermore, this particular theoretical perspective predicts overlap both for movements of the body and action-effects in the environment. While studies demonstrated imitation of perceived action-effects produced by another person (see the research of Bekkering et al. (2000) above), research on socio-evaluative consequences of imitation exclusively studied imitation of perceived movements. Therefore, the present study asked whether imitation of the action-effects of another person’s action *without* imitation of her movements would affect the social-affective evaluation of this person.

We conducted three experiments to investigate whether imitation of action-effects would affect the social affiliation with another person. Experiment 1 represented a proof of principle for the design of our study. Participants first acquired action-effect associations in a free-choice training phase. In a subsequent test phase, participants produced visual action-effects with bimanual vertical and horizontal movements following the observation of videotaped models who also performed movements producing visual action-effects. Critically, movement and action effect of the model could either be similar or different to the participants own movement and action-effect. The main focus of this research is on the social-affective consequences that imitation or counter-imitation of action-effects might have. To measure socio-evaluative effects, we asked participants after each trial to judge how much they felt affiliated with the observed model and used these subjective ratings as the main dependent variable for analysis. For completeness, results of performance data are presented in the supplement (although the present experiments were not designed to assess motoric imitation effects).

We used different instructions in Experiments 1 and 2 to manipulate whether participants would represent the action in terms of an intended body movement or in terms of an intended action effect, respectively (for similar manipulations see Ansorge, 2002; Hommel, 1993; Zwosta, Ruge, & Wolfensteller, 2013). In Experiment 1, we highlighted body movements in the task instructions and expected to replicate previous findings showing that performing a similar (relative to a dissimilar) movement as another person increases social affiliation ratings for this person. In Experiment 2, we instructed actions in terms of their action-effects and predicted that producing a similar action-effect as another person increases social affiliation ratings for this person relative to dissimilar action-effects. In Experiment 3, participants produced action-effects with key presses on a keyboard while the videos showed bimanual horizontal and vertical movements as in Experiment 1 and 2. The difference in the input devices should have eliminated a similarity on the kinematic movement level. We hypothesized that observations of similar action-effects would increase social affiliation towards the other person (relative to observation of dissimilar action-effect), even when these effects were produced with physically dissimilar movements.

## Experiment 1

**Fig. 1:**
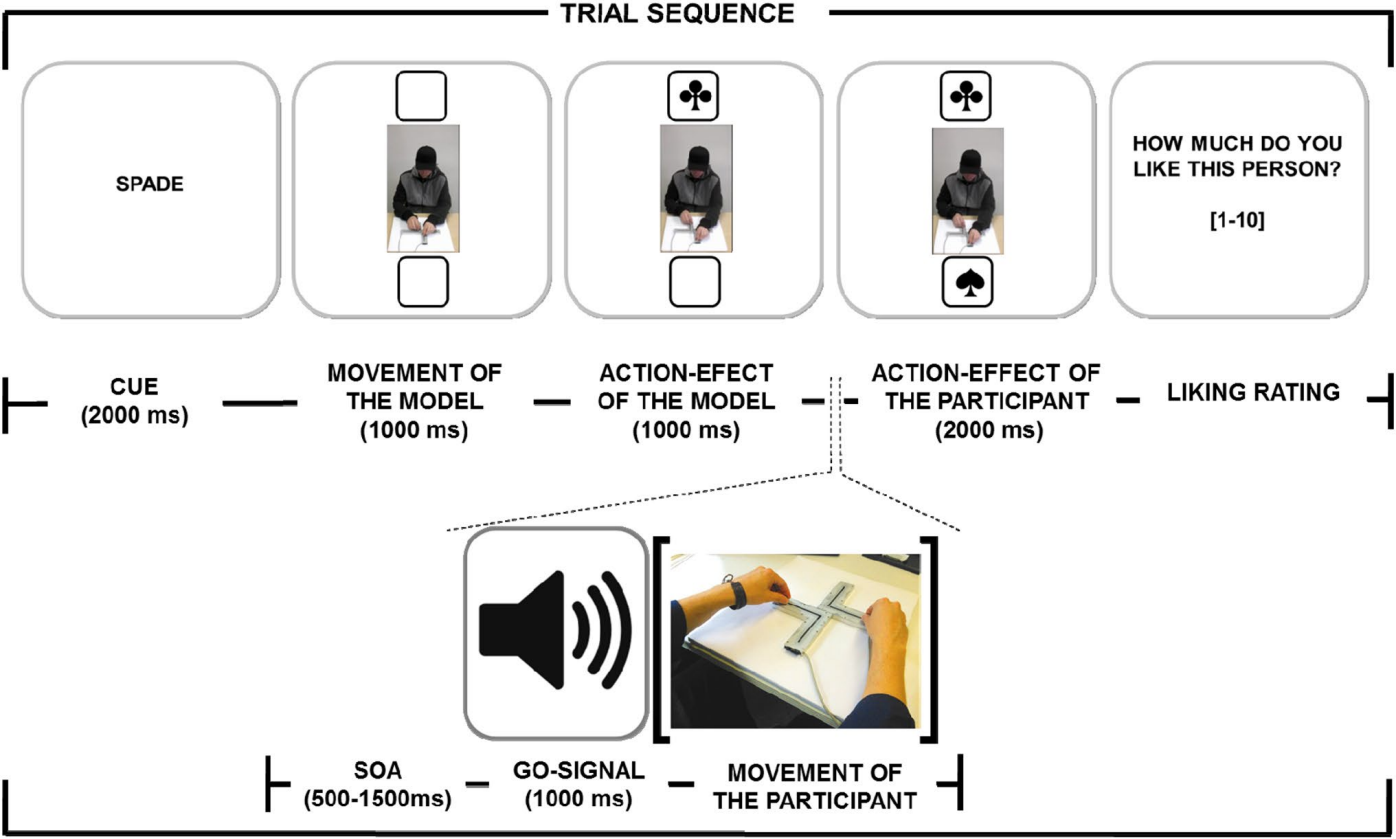
Sequence of trial events in Experiment 2. Participants observed a video of a model performing a vertical or horizontal bimanual movement with a slide control, followed by the presentation of an action-effect of the model (e.g., in the upper location on the screen). Then a go-signal indicated that participants should perform the cued movement with the slide control, which produced an action-effect of the participants (e.g., in the lower location of the screen). At the end of the trial participants provided a social affiliation rating for the model with an external key pad. Participants were instructed to attend the movements of the model in Experiment 1 whereas they were instructed to attend to the action effect produced by the model in Experiment 2

Participants viewed video clips in which several models performed two distinctive bimanual arm movements that were clearly distinguishable for the observer. Each movement was followed by a visual action-effect. After each video clip, participants were instructed to carry out a specific bimanual arm movement that triggered a visual action-effect. We manipulated movement compatibility and effect compatibility orthogonally. That is: (1) arm movements of the model and the participant and (2) ensuing (action-) effects of the model and the participants were either similar (compatible) or dissimilar (incompatible). A typical trial sequence is depicted in Fig. 1. To assess the influence of movement and effect compatibility on social-affective evaluations, participants rated the affiliation with the model at the end of each trial. Task instructions highlighted to pay attention to the movements performed by the model.

## Method

### Participants

Based on previous research from our lab that investigated imitation effects on social affiliation (e.g., *d*_z_ = 0.61 for Exp. 1 of Dignath et al., 2018), we planned to recruit about 30 participants for both Experiment 1 and 2 to aim for a power of 1 − *β* = 0.90 for this effect size (two-sided *t* test, alpha = 0.05). We, therefore, recruited 31 participants (3 left-handers, 31 women, *M* = 23.8 years, range 19–54 years) who either received course credit or a monetary reimbursement. Participants were naive regarding the purpose of the experiment. Exclusion criteria were identical for all experiments: participants with more than 50% error rate were removed due to random performance in the two-alternative forced-choice task. From the remaining sample, all participants with a mean error rate above 2.5 SDs were treated as outliers.^1^ Data of one participant were excluded due to unusual high error rates in Experiment 1 (*M* > 25%, > 2.5 SDs). Raw data and analysis scripts for the reported experiments can be retrieved from https://osf.io/mjw4g/.

### Apparatus and stimuli

To make sure that participants could distinguish the movements easily, a slide control was used which consisted of two rectangular metal brackets that were fixed to a wooden board forming a cross (see the photograph in Fig. 1; see also Dignath et al., 2018). A plastic knob was attached on top of each bracket allowing either a horizontal (left vs. right) or a vertical movement (towards vs. away from the controller). Movement data were collected by photoelectric barriers at each start and end position of the slide control.

To create short movie clips, 40 volunteers were videotaped (27 women). Each model was seated in front of a neutral background behind a desk, leaving only the upper body part visible. Each model wore the same dark jacket and a black cap and the eyes of the model were concealed by a combination of a high-angle perspective of the camera and a slightly tilted head posture. This method of de-individualization was meant to reduce error variance due to the physical attractiveness of the models (cf. Sparenberg et al., 2012). Using the slide control that was placed on top of the desk, two videotapes were recorded with each model. In both videos, the starting position of the knobs was in the middle of the cross. In one video clip, the model moved the right knob with the right hand to the right end of the cross and synchronously the left knob with the left hand to the left end of the cross (horizontal movement). The second clip showed the opposite action by taping the actor pushing the left knob away from their position while again in a synchronous manner pulling the right knob towards them (vertical movement). To control the speed of the model’s movements a metronome (https://www.metronomeonline.com) synchronized the model’s behavior at a constant pace of 44 beats-per-minute. Each video clip had a length of 1 s and was muted to eliminate the metronome sound (an example movie clip [not used in the experiment] is available in the OSF project). Movie clips were pre-rated by 33 neutral raters on a 0–9 rating scale using paper–pencil according to attractiveness (*M* = 3.46, SD = 0.58) and affiliation (*M* = 3.04, SD = 0.56) of the person shown. Please note that this scale and the response mode differed from the reported experiments.

The experiment was conducted using a professional software timer and presented on a 17 inch monitor. Videos were presented at the center of the screen with a width of 162 pixels and a height of 288 pixels. Action-effects were presentations of a colored (yellow/turquois/purple) square after a movement that were presented in a separate frame of 80 × 80 pixels either above or below the video showing the model (counterbalanced position across participants, see design section). Auditory cues were presented binaurally via Philips SHP2000 headphones. Social affiliation ratings were collected using an external numeric keypad.

### Procedure

The experiment consistent of two phases: a training phase and a test phase.

#### Training

In a first training phase, participants learned to execute the movements using the slide control and to associate each movement with a unique colored action-effect. At the beginning of the training participants were informed about the two relevant types of movement (“Vertical: You pull the knobs to the top and to the bottom”. and “Horizontal: You pull the knobs to the right and to the left”.). Participants could freely decide which movement to perform on each training trial. Instructions also stated that they should choose each movement approximately equal times throughout the training phase without using a systematic response strategy (e.g. alternating on every trial). If participants committed an error during the first training trials, they were verbally corrected by the instructor. The training phase finished after participants had correctly performed each movement at least ten times.

#### Test

At the beginning of the test phase, participants were informed that for the rest of the experiment, they had to perform only one of the two movement types (counterbalanced across participants). Instructions referred explicitly to the movement, and did not make explicit mention of the action effect:

#### “Please execute the following movement: horizontal (vertical)”

Furthermore, they were informed that on each trial they would see the video of another person and that they should rate for each person how much they liked the person shown in the video. Each trial started with an exclamation mark presented in the middle of the screen for 2 s, followed by a 1 s blank screen. Then, a video with the model’s movement (horizontal/vertical) was presented for 1 s, followed by the action-effect of the model, presented in the upper or lower frame for 1.5 s. Both the last frame of the video and the action-effect of the model remained on the screen until the end of the movement task. After a variable stimulus onset asynchrony (SOA: 0.5–1.5 s) a go-signal (800 Hz tone) was played for 1 s, indicating that participants had to carry out the instructed movement as precisely and quickly as possible. If participants did not start the action within 1 s or took longer than 2 s to complete the movement, an error message was shown. Similarly, an error message was presented for trials in which the two knobs were not moved in synchrony (i.e., if the first knob arrived at the end position before the second knob had left the starting position).

Correct movements produced an action effect presented at the designated location in the upper or lower frame for 2 s. The movement (horizontal/vertical) to action-effect (purple/yellow square) mapping was randomized across participants. The action-effect generated by the other movement, executed only in the training not in the test phase, was always a turquoise square. In addition, the location (frame) at which action-effects of the model and the participant were presented was counterbalanced across participants. At the end of the trial, a text box was presented, asking the participant to indicate on a 10-point scale how much they liked the person in the video (1 = not at all; 10 = very much) by entering a digit on the key pad. Eventually, participants were asked to return the knob to the starting position. Each participant went through 40 trials. Participants were exposed to every model only once in a randomized order. Four sets of ten videos each were created and assigned to each condition (randomized across participants).

### Design

A 2 × 2 within-design was used to manipulate (1) whether participants performed the same or a different movement as the model and (2) whether participants produced the same or a different action-effect as the model. To assess how these factors influence social-affective evaluations, participants provided explicit ratings of each model at the end of a trial.

## Results

### Data selection and analyses

Test trials with response anticipations (2.6%), non-synchronous responses where the first knob had reached the end position before the second knob had left the starting position (0.8%), responses slower than 1000 ms after onset of the go signal and/or with movement times exceeding 2 s (3.1%), and erroneous responses (1.5%) were discarded from the analysis. Mean scores of social affiliation ratings were calculated for each design cell and analyzed with an analysis of variance (ANOVA) with the within-factors *movement compatibility* (same vs different movements of model and participant) and *effect compatibility* (same vs different action-effects of model and participant).

Furthermore, we performed analyses of participants’ performance data, that is initiation times (IT), movement times (MT) and error rates with the same ANOVA model as used for social affiliation ratings (Supplementary Material). Please note that these analyses are exploratory, since our design was not optimized to assess motoric imitation effects and we present performance data here only for completeness (see Table 1 for descriptive data for all three experiments).

**Table 1:** Means and standard error of the mean in parenthesis for correct initiation times (IT), movement times (MT) and error rates in the experiments as a function of movement compatibility and (action-) effect compatibility

	IT (ms)				MT (ms)				Error (%)			
Action-effect	Inc	Inc	Com	Com	Inc	Inc	Com	Com	Inc	Inc	Com	Com
Movement	Inc	Com	Inc	Com	Inc	Com	Inc	Com	Inc	Com	Inc	Com
Experiment 1	490 (23)	489 (24)	498 (21)	473 (22)	487 (24)	483 (24)	478 (24)	481 (24)	1.74 (72)	1.37 (0.65)	1.74 (0.72)	1.37 (0.65)
Experiment 2	647 (30)	647 (25)	644 (27)	630 (28)	496 (33)	489 (31)	501 (32)	497 (34)	3.19 (1.66)	3.71 (0.98)	2.90 (1.04)	3.30 (1.07)
Experiment 3	649 (65)	–	695 (69)	–	–	–	–	–	10.91 (1.56)	–	7.91 (1.28)	–

*Com* compatible, *inc* incompatible

### Social affiliation ratings

**Fig. 2:**
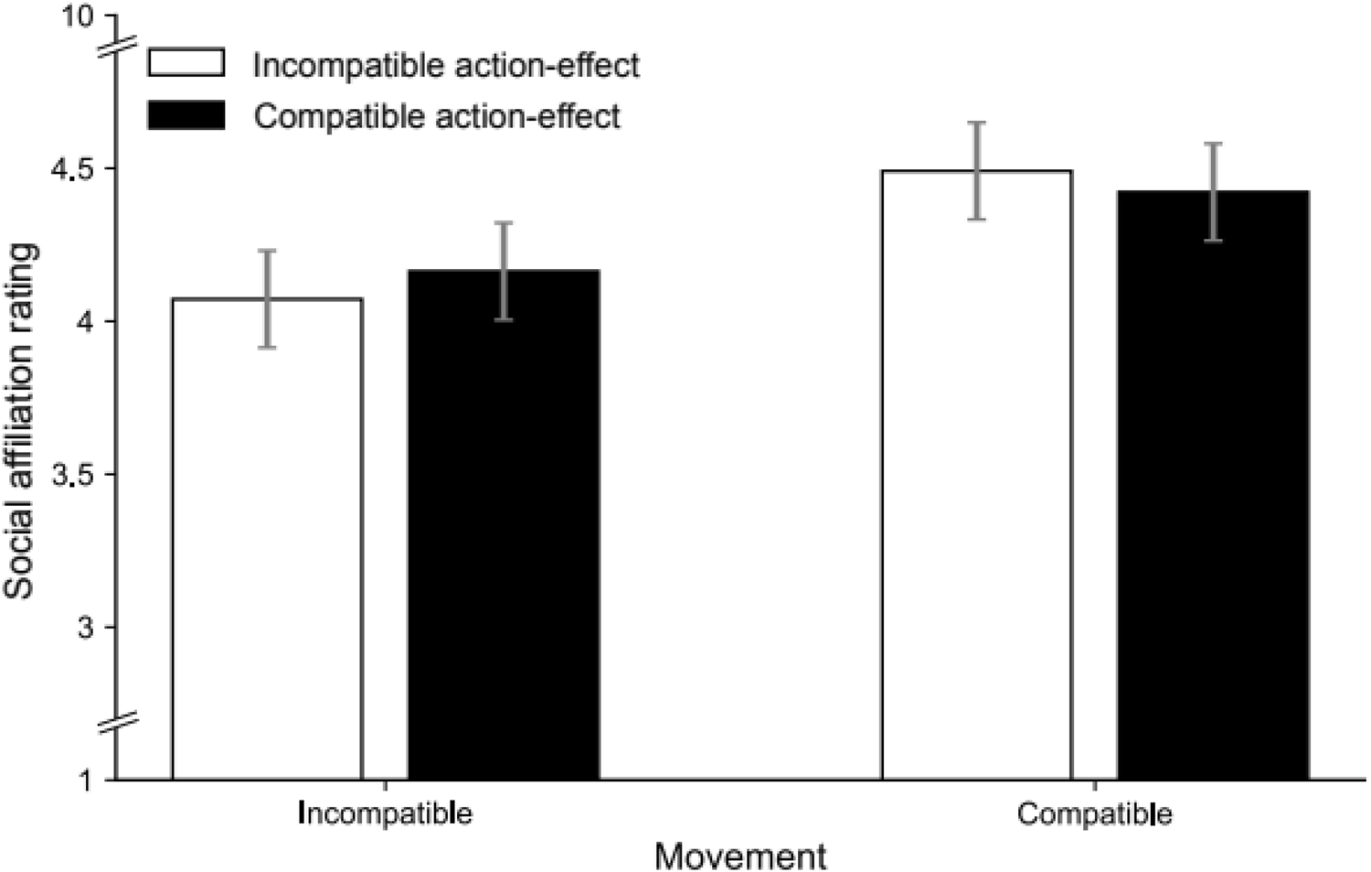
Mean social affiliation ratings of models for the different movement compatibility (different [left] vs. same [right] movements as the model) and (action-) effect compatibility (different [white] vs. same [black] effects as the model) conditions of Experiment 1. Error bars show the standard error of the mean for the paired differences between the ‘movement compatible’ and ‘movement incompatible’ condition

Figure 2 shows the mean ratings for each condition. The ANOVA returned a significant main effect of movement compatibility, *F*(1, 29) = 4.76, *p* = 0.037, $\eta_{p}^{2}$ = 0.141, showing that participants rated models with the same movements as more positive (*M* = 4.46, SD = 0.99) than models with different movements (*M* = 4.11, SD = 1.11). Effect compatibility did not influence ratings, *F* < 1, and the interaction between both factors was not significant either, *F*(1, 29) = 1.03, *p* = 0.318, $\eta_{p}^{2}$ = 0.034. These results validate our experimental design by showing the hypothesized effect of imitation on social affiliation.

## Experiment 2

Instructions in Experiment 1 highlighted a representation of the imitated action in terms of body movements. Experiment 2 examined whether analogous social-affective consequences would be obtained with the imitation of action-effects. Therefore, instructions for this experiment emphasized the action effect that should be produced with a particular slider movement. In addition, participants had to select one of two possible movements/action-effects on each trial which should increase the relevance of the action effect for the movement task.

## Method

### Participants

Thirty-two students at the University of Würzburg (all right handers, 32 women, *M* = 26.0 years, range 19–38 years) were paid for participation. Participants were naive regarding the purpose of the experiment. Data of two participants were excluded due to unusually high error rates (*M* > 48%, > 2.5 SDs).

### Apparatus and stimuli

Stimuli were identical to Experiment 1, except that we replaced color patches as action-effects stimuli with a black and white symbol of a spade or a clubs (same size as in Experiment 1).

### Procedure and design

The procedure differed from Experiment 1 in two aspects. First, task instructions now asked participants to produce a specific action-effect: “Please produce a spade (clubs) on the screen”. Second, participants performed both movements (producing both action-effects) within the same test session. Task instructions specified at the beginning of each trial which action effect should be produced. The same within-subjects design as in Experiment 1 was used.

## Results

### Data selection and analyses

Test trials with response anticipations (4.3%), non-synchronous responses (0.9%), slow responses (1.3%), and erroneous responses (3%) were discarded from the analysis. Mean scores of social affiliation rating were analyzed with the same ANOVA as in Experiment 1. For descriptive performance data, see Table 1.

### Social affiliation ratings

**Fig. 3:**
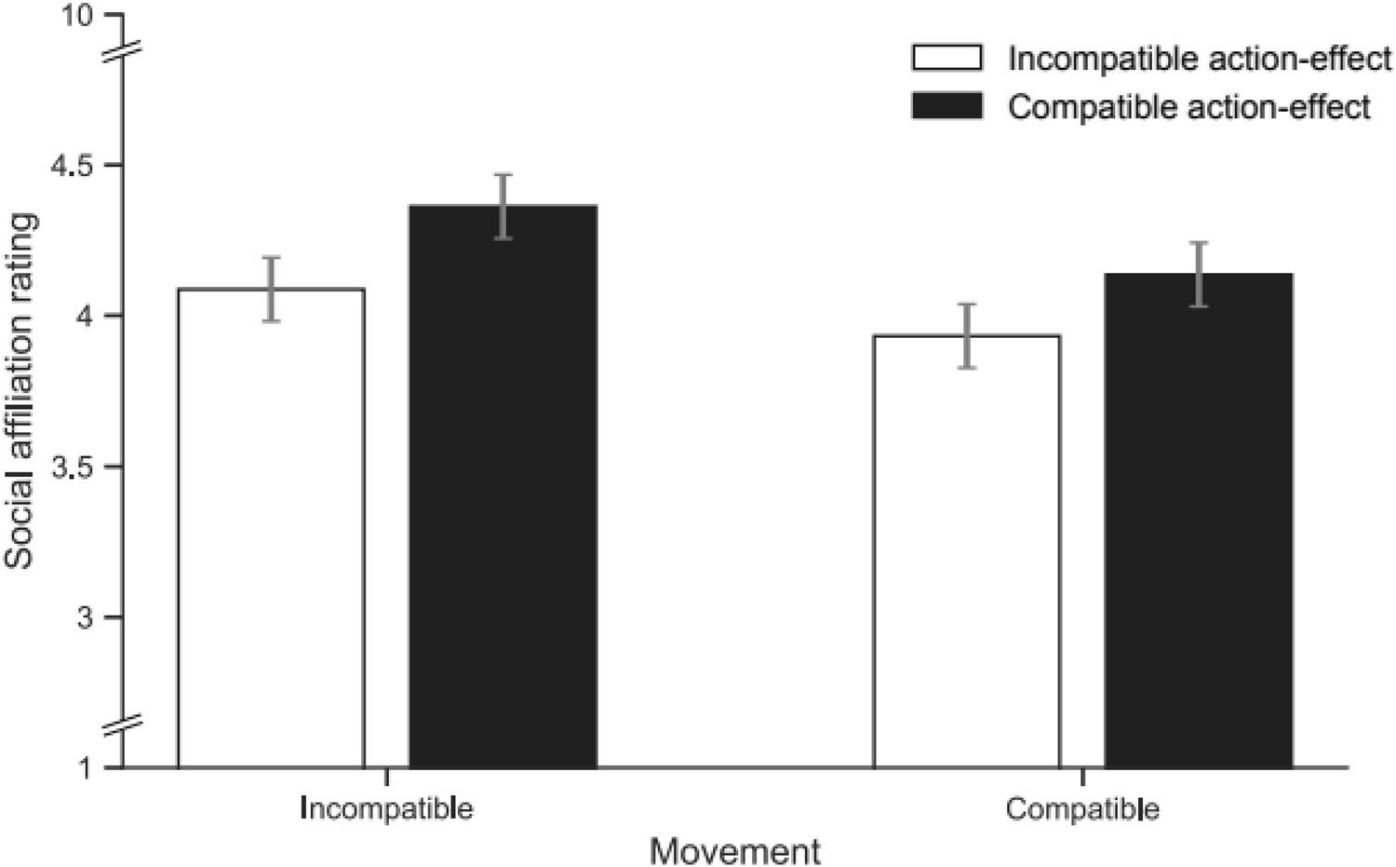
Mean social affiliation ratings of models for the different movement compatibility (different [left] vs. same [right] movements as the model) and (action-) effect compatibility (different [white] vs. same [black] effects as the model) compatibility conditions of Experiment 2. Error bars show the standard error of the mean for the paired differences between the ‘action-effect compatible’ and ‘action-effect incompatible’ condition

Figure 3 shows the mean ratings for each condition. A main effect of effect compatibility showed that participants rated models producing the same action effects as more positive (*M* = 4.25, SD = 1.41) than models producing different action effects (*M* = 4.01, SD = 1.27), *F*(1, 29) = 5.38, *p* = 0.028, *η*_*p*_^*2*^ = 0.157. In contrast, similarity of the movements performed by the models and participants had no effect on the rating, *F*(1, 29) = 1.42, *p* = 0.243, $\eta_{p}^{2}$ = 0.047. The interaction was also not significant, *F* < 1, suggesting that the altered action representation in terms of action-effects in the environment now determined the influence of imitation on social affiliation.

## Experiment 3

In Experiment 3, we aimed to replicate and generalize this finding to a situation in which any movement compatibility is eliminated. Participants, therefore, observed the same videos as before but responded with key presses on a keyboard, instead of moving the slide control (as the models in the video). This should render executed and observed movement very dissimilar in terms of the underlying kinematics of the movements.

### Method

#### Participants

A power analysis performed with G*Power 3 (Faul, Erdfelder, Lang, & Buchner, 2007) suggested a sample size of *N* = 48 to replicate the effect of effect compatibility of *d*_*z*_ = 0.43 observed in Experiment 2 (with *α* = 0.05 and 1 − *β* = 0.90 in a one-tailed *t* test). Fifty-nine participants (9 left-handers, 42 women, *M* = 25.4 years [age of one participant was not recorded], range 16–62 years) were paid for taking part in the experiment. Participants were naive regarding the purpose of the study. Data of four participants were excluded due to high error rates (> 50%).

#### Apparatus and procedure

Stimuli were identical to Experiment 2. Instead of using a slide control, participants produced action-effects with key presses of the keys ‘D’ and ‘L’ on a QWERTZ keyboard using the index fingers of both hands. The mapping of the keys to the action-effects was counterbalanced across participants. The procedure was identical to Experiment 2.

## Results

**Fig. 4:**
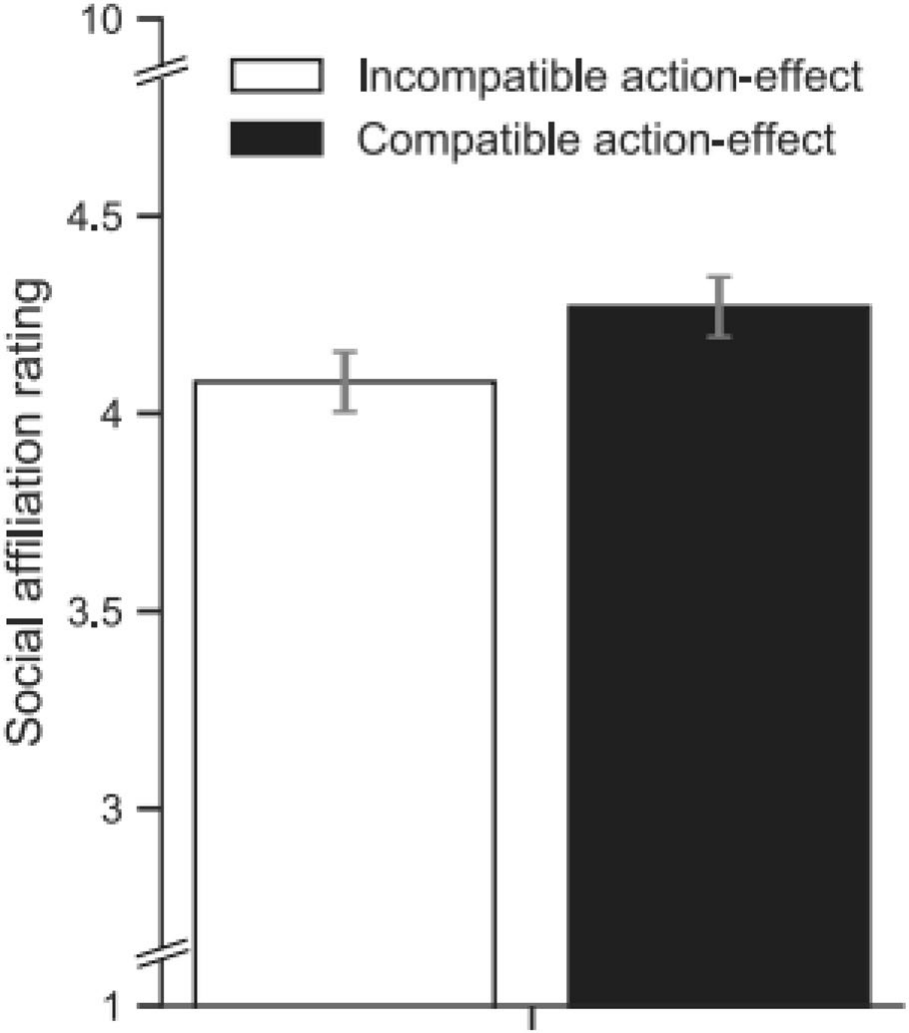
Mean social affiliation ratings of models for the (action-) effect compatibility (different [white] vs. same [black] effects as the model) condition of Experiment 3. Error bars show standard error of the mean for the paired differences between the ‘action-effect compatible’ and ‘action-effect incompatible’ condition

Test trials with erroneous responses (9.4%) were discarded from the analysis, no response anticipations or omissions occurred during the experiment. Descriptive performance data are displayed in Table 1. Mean social affiliation ratings were analyzed with a *t* test for paired samples. As shown in Fig. 4, participants rated models that produced the same action-effects as more positive (*M* = 4.27, SD = 1.4) than models that produced different action-effects (*M* = 4.08, SD = 1.4), *t *(54) = 2.41, *p* = 0.019, *d*_*z*_ = 0.33.

## General discussion

The present study examined whether producing similar action effects as another person increases liking of that person, even when the observed and performed movements were different. Results of Experiment 1 showed that participants evaluated models more positively after they had performed the same movements compared to different movements. This replicates previous research showing that imitation of body movements results in a more positive social-affective evaluation of the imitated person (Catmur & Heyes, 2013; de Coster et al., 2013; Dignath et al., 2018). It also shows that our experimental paradigm was a valid approach to investigate the influence of imitation on social-affective evaluations. In Experiment 2, instructions framed the action of participants in terms of the action-effect and not the movement. Results showed that participants liked models who produced similar action-effects more than models who produced different action effects—irrespective of the type of body movement that was necessary to produce these effects. In Experiment 3, models and participants performed distinct movement sets, ruling out a perceptual similarity of hand movements on the element level. The experiment reproduced the effect of effect compatibility on liking ratings: participants provided more positive evaluations for models who produced the same action-effect relative to models producing different action-effects.

Together, these results are in line with effect-based accounts of action control, such as the ideomotor framework, that explain the effect of observed actions on self-generated actions with a common coding of actions in terms of intended action-effects (Hommel, 2013; Prinz, 2002). They further support the notion that agents represent their actions in terms of the perceived action-effects, which can be either the movement of the body or the consequence of this movement in the environment (Pfister, 2019). Crucially, the present research extends the ideomotor framework by showing that imitation of others’ action-effects not only has behavioral, but also social, consequences.

### Imitation of movement or action effect?

It is currently debated which aspect of an action is most relevant for performance effects of imitation (see Cracco et al., 2018), with some studies arguing for movement parameters as the major source (e.g., Bird, Brindley, Leighton, & Heyes, 2007; Genschow, Florack, & Waenke, 2013; Cole, Atkinson, D'Souza, Welsh, & Skarratt, 2018), while others found support for action-effects as the more relevant source for imitation (e.g., Bekkering, Wohlschläger, & Gattis, 2000; Bouquet, Shipley, Capa, & Marshall, 2011). To account for the diverging findings, Heyes and colleagues suggested that the relative salience of movements and action effects determines which action component becomes more relevant for motor imitation (see Bird, Brindley, Leighton, & Heyes, 2007; Leighton, Bird, & Heyes, 2010). Theoretically, saliency could modulate how stimulus–response codes are weighted in a task-set (Memelink & Hommel, 2013; Frings et al., 2020). One way to manipulate saliency top-down are instructions. In line with a relative saliency account, Experiment 1 and 2 showed that increased social affiliation for imitation depended on the instruction that was given for the action task. If the action was framed in terms of the intended movement, similar movements, and not action effects, increased social affiliation; if the action was framed in terms of intended action effects, action-effects, and not movements, had an effect on social affiliation. However, the present experiments were not designed to provide a test of the relative saliency account, and we acknowledge that Experiment 1 and 2 differed in several aspects, which limits the interpretation of such a between-experiment comparison. Future research could test these ideas more directly by comparing both instructions within a single experiment.

### Social-affective consequences of action-effect imitation: mechanisms

Important questions remain. Specifically, a better understanding of the underlying mechanisms how similar action-effects lead to increased social affiliation is needed. One possibility is that processing dynamics elicit an affective response that provides the basis for social evaluation. Support for this speculation comes from research on fluency (Winkielman & Cacioppo, 2001), showing that repetition of perceived stimuli (e.g., Reber, Winkielman, & Schwarz, 1998) or executed movements (e.g., Hayes, Paul, Beuger, & Tipper, 2008) is closely linked to positive affect. In the context of the present research fluency could result from anticipation of own action-effects, which is assumed to rely on perceptual codes (see James, 1980; Hommel et al., 2001), and perception of similar action-effects for the other person. A different explanation is that similar action-effects provide a cue for social categorization. In line with research on group identification (Tajfel, Billig, Bundy, & Flament, 1971; for reviews, see Otten, 2016; Dunham, 2018), participants could have used the similarity of action-effects to infer group membership. According to this account, affective responses are indirect, in the sense that participants would tend to evaluate in-group members more positively than out-group members. A related question is whether affective response or social categorization due to similar action-effects requires awareness. While a rich research tradition suggests that many social cognitive processes (including mimicry effects) can occur outside of participants’ awareness (see Bargh, Schwader, Hailey, Dyer, & Boothby, 2012; for mimicry, see Chartrand & Lakin, 2013). An alternative account would assume that awareness of ‘sharing’ the same action-effect with another person allows participants to draw inferences about other people’s mental states and thereby increases social affiliation (e.g., Lakens & Stel, 2011). In short, several processes (fluency, automatic mimicry, inferential reasoning) can account for the present results that should be examined in future research.

## Summary

This present research provided first evidence that the imitation of action effects of another person increases social affiliation, even when the performed movements were different. This finding supports the ideomotor framework and points to a possible role of action-effects for action planning in social interaction beyond motor coordination (for a discussion see Kunde, Weller, & Pfister, 2018). In fact, the function of shared action-effects as a ‘social glue’ to foster cooperation among people might be particular relevant in many everyday joint actions (e.g., carrying a table together) for which agents often perform different (complementary) movements to achieve a common action effect.

## Electronic supplementary material

Below is the link to the electronic supplementary material.

Supplementary file1 (DOCX 13 kb)

## Data Availability

The datasets generated and analyzed during the current study are available in the Open Science Framework repository, https://osf.io/mjw4g/. 10.17605/OSF.IO/MJW4G.

## Footnotes

1 Including outlier data in the analysis did not change the level of significance for the reported results.

## References

[CR1] Ansorge U (2002). Spatial intention–response compatibility. Acta Psychologica.

[CR2] Bargh JA, Schwader KL, Hailey SE, Dyer RL, Boothby EJ (2012). Automaticity in social-cognitive processes. Trends in Cognitive Sciences.

[CR3] Bekkering H, Wohlschlager A, Gattis M (2000). Imitation of gestures in children is goal-directed. The Quarterly Journal of Experimental Psychology: Section A.

[CR4] Bird G, Brindley R, Leighton J, Heyes C (2007). General processes, rather than "goals", explain imitation errors. Journal of Experimental Psychology: Human Perception and Performance.

[CR5] Bouquet CA, Shipley TF, Capa RL, Marshall PJ (2011). Motor contagion: goal-directed actions are more contagious than nongoal-directed actions. Experimental Psychology.

[CR6] Brass M, Bekkering H, Wohlschläger A, Prinz W (2000). Compatibility between observed and executed finger movements: Comparing symbolic, spatial, and imitative cues. Brain and Cognition.

[CR7] Brass M, Heyes C (2005). Imitation: Is cognitive neuroscience solving the correspondence problem?. Trends in Cognitive Sciences.

[CR8] Catmur C (2016). Automatic imitation? Imitative compatibility affects responses at high perceptual load. Journal of Experimental Psychology: Human Perception and Performance.

[CR9] Catmur C, Heyes C (2013). Is it what you do, or when you do it? The roles of contingency and similarity in pro-social effects of imitation. Cognitive Science.

[CR10] Chartrand TL, Bargh JA (1999). The Chameleon effect: The perception–behavior link and social interaction. Journal of Personality and Social Psychology.

[CR11] Chartrand TL, Lakin JL (2013). The antecedents and consequences of human behavioral mimicry. Annual Review of Psychology.

[CR12] Cole GG, Atkinson MA, D'Souza AD, Welsh TN, Skarratt PA (2018). Are goal states represented during kinematic imitation?. Journal of Experimental Psychology: Human Perception and Performance.

[CR13] Colton J, Bach P, Whalley B, Mitchell C (2018). Intention insertion: Activating an action’s perceptual consequences is sufficient to induce non-willed motor behavior. Journal of Experimental Psychology: General.

[CR14] Cracco E, Bardi L, Desmet C, Genschow O, Rigoni D, De Coster L, Brass M (2018). Automatic imitation: A meta-analysis. Psychological Bulletin.

[CR15] De Coster L, Verschuere B, Goubert L, Tsakiris M, Brass M (2013). I suffer more from your pain when you act like me: Being imitated enhances affective responses to seeing someone else in pain. Cognitive, Affective, & Behavioral Neuroscience.

[CR16] Dignath D, Eder AB (2013). Recall of observed actions modulates the end-state comfort effect just like recall of one’s own actions. Experimental Brain Research.

[CR17] Dignath D, Kiesel A, Frings C, Pastötter B (2020). Electrophysiological evidence for action-effect prediction. Journal of Experimental Psychology: General.

[CR18] Dignath D, Lotze-Hermes P, Farmer H, Pfister R (2018). Contingency and contiguity of imitative behaviour affect social affiliation. Psychological Research Psychologische Forschung.

[CR19] Dignath D, Pfister R, Eder AB, Kiesel A, Kunde W (2014). Representing the hyphen in action–effect associations: Automatic acquisition and bidirectional retrieval of action–effect intervals. Journal of Experimental Psychology: Learning, Memory, and Cognition.

[CR20] Dunham Y (2018). Mere membership. Trends in Cognitive Sciences.

[CR21] Elsner B, Hommel B (2001). Effect anticipation and action control. Journal of Experimental Psychology: Human Perception and Performance.

[CR22] Faul F, Erdfelder E, Buchner A, Lang AG (2009). Statistical power analyses using G* Power 3.1: Tests for correlation and regression analyses. Behavior research methods.

[CR23] Frings C, Hommel B, Koch I, Rothermund K, Dignath D, Giesen C, Kiesel A, Kunde W, Mayr S, Moeller B, Möller M, Pfister R, Philipp A (2020). Binding and retrieval in action control (BRAC). Trends in Cognitive Science.

[CR24] Genschow O, Florack A, Wänke M (2013). The power of movement: Evidence for context-independent movement imitation. Journal of Experimental Psychology: General.

[CR25] Greenwald AG (1970). Sensory feedback mechanisms in performance control: With special reference to the ideo-motor mechanism. Psychological Review.

[CR26] Hale J, Hamilton AFDC (2016). Cognitive mechanisms for responding to mimicry from others. Neuroscience & Biobehavioral Reviews.

[CR27] Hayes AE, Paul MA, Beuger B, Tipper SP (2008). Self produced and observed actions influence emotion: The roles of action fluency and eye gaze. Psychological Research Psychologische Forschung.

[CR28] Heyes C (2001). Causes and consequences of imitation. Trends in Cognitive Sciences.

[CR29] Heyes C (2011). Automatic imitation. Psychological Bulletin.

[CR30] Heyes C (2018). Empathy is not in our genes. Neuroscience and Biobehavioral Reviews.

[CR31] Hommel B (1993). Inverting the Simon effect by intention. Psychological Research Psychologische Forschung.

[CR32] Hommel B, Prinz W, Beisert M, Herwig A (2013). Ideomotor action control: On the perceptual grounding of voluntary actions and agents. Action science: Foundations of an emerging discipline.

[CR33] Hommel B, Müsseler J, Aschersleben G, Prinz W (2001). The theory of event coding (TEC): A framework for perception and action planning. Behavioral and Brain Sciences.

[CR34] Inzlicht M, Gutsell JN, Legault L (2012). Mimicry reduces racial prejudice. Journal of Experimental Social Psychology.

[CR35] James W (1890). The principles of psychology.

[CR36] Jansson E, Wilson AD, Williams JH, Mon-Williams M (2007). Methodological problems undermine tests of the ideo-motor conjecture. Experimental Brain Research.

[CR37] Kilner JM, Friston KJ, Frith CD (2007). Predictive coding: An account of the mirror neuron system. Cognitive Processing.

[CR38] Kunde W (2003). Temporal response-effect compatibility. Psychological Research.

[CR39] Kühn S, Keizer AW, Rombouts SA, Hommel B (2011). The functional and neural mechanism of action preparation: Roles of EBA and FFA in voluntary action control. Journal of Cognitive Neuroscience.

[CR40] Kunde W, Weller L, Pfister R (2018). Sociomotor action control. Psychonomic Bulletin & Review.

[CR41] Lakens D, Stel M (2011). If they move in sync, they must feel in sync: Movement synchrony leads to attributions of rapport and entitativity. Social Cognition.

[CR42] Leighton J, Heyes C (2010). Hand to mouth: Automatic imitation across effector systems. Journal of Experimental Psychology: Human Perception and Performance.

[CR43] Memelink J, Hommel B (2013). Intentional weighting: A basic principle in cognitive control. Psychological Research Psychologische Forschung.

[CR44] Ondobaka S, de Lange FP, Newman-Norlund RD, Wiemers M, Bekkering H (2012). Interplay between action and movement intentions during social interaction. Psychological Science.

[CR45] Otten S (2016). The Minimal Group Paradigm and its maximal impact in research on social categorization. Current Opinion in Psychology.

[CR46] Pfister R (2019). Effect-based action control with body-related effects: Implications for empirical approaches to ideomotor action control. Psychological Review.

[CR47] Pfister R, Dignath D, Hommel B, Kunde W (2013). It takes two to imitate: Anticipation and imitation in social interaction. Psychological Science.

[CR48] Pfister R, Janczyk M, Gressmann M, Fournier LR, Kunde W (2014). Good vibrations? Vibrotactile self-stimulation reveals anticipation of body-related action effects in motor control. Experimental Brain Research.

[CR49] Pfister R, Janczyk M, Wirth R, Dignath D, Kunde W (2014). Thinking with portals: Revisiting kinematic cues to intention. Cognition.

[CR50] Pfister R, Kunde W (2013). Dissecting the response in response–effect compatibility. Experimental Brain Research.

[CR51] Powers WT (1973). Behavior: The control of perception.

[CR52] Prinz, W. (2002). Experimental approaches to imitation. In A. N. Meltzoff & W. Prinz (Eds.), *The imitative mind: development, evolution, and brain bases* (pp. 143–162). New York: Cambridge University Press.

[CR53] Prinz J, Hurley S, Chater N (2005). Imitation and moral development. Perspectives on imitation: From neuroscience to social science.

[CR54] Reber R, Winkielman P, Schwarz N (1998). Effects of perceptual fluency on affective judgments. Psychological Science.

[CR55] Romanes GJ (1882). Animal intelligence.

[CR56] Shin YK, Proctor RW, Capaldi EJ (2010). A review of contemporary ideomotor theory. Psychological Bulletin.

[CR57] Sparenberg P, Topolinski S, Springer A, Prinz W (2012). Minimal mimicry: Mere effector matching induces preference. Brain and Cognition.

[CR58] Tajfel H, Billig MG, Bundy RP, Flament C (1971). Social categorization and intergroup behaviour. European Journal of Social Psychology.

[CR59] Van Baaren RB, Holland RW, Kawakami K, Van Knippenberg A (2004). Mimicry and prosocial behavior. Psychological Science.

[CR60] Van Baaren RB, Holland RW, Steenaert B, van Knippenberg A (2003). Mimicry for money: Behavioral consequences of imitation. Journal of Experimental Social Psychology.

[CR61] van Steenbergen H, Warren CM, Kühn S, de Wit S, Wiers RW, Hommel B (2017). Representational precision in visual cortex reveals outcome encoding and reward modulation during action preparation. NeuroImage.

[CR62] Whiten A, Ham R (1992). On the nature and evolution of imitation in the animal kingdom: Reappraisal of a century of research. Advances in the Study of Behavior.

[CR63] Winkielman P, Cacioppo JT (2001). Mind at ease puts a smile on the face: Psychophysiological evidence that processing facilitation elicits positive affect. Journal of Personality and Social Psychology.

[CR64] Wohlschläger A, Bekkering H (2002). Is human imitation based on a mirror-neurone system? Some behavioural evidence. Experimental Brain Research.

[CR65] Wohlschläger A, Gattis M, Bekkering H (2003). Action generation and action perception in imitation: An instance of the ideomotor principle. Philosophical Transactions of the Royal Society of London. Series B: Biological Sciences.

[CR66] Zwosta K, Ruge H, Wolfensteller U (2013). No anticipation without intention: Response–effect compatibility in effect-based and stimulus-based actions. Acta Psychologica.

