# Supplementary material for: Imitation of action-effects increases social affiliation
Source: Psychol Res. 2020 Jul 14;85(5):1922–33. doi: 10.1007/s00426-020-01378-1 (PMC8289777; doi:10.1007/s00426-020-01378-1)
Supplement: Supplementary file 1 — Supplementary file1 (DOCX 13 kb) [file 426_2020_1378_MOESM1_ESM.docx]

**Supplementary material “Imitation of action-effects increases social affiliation”**

**Exploratory Analysis of performance Data**

We performed analyses of participants’ performance data, that is initiation times (IT), movement times (MT) and error rates with the same ANOVA/ t-test model as used for social affiliation ratings (main text). Please note that these analysis are exploratory, since our design was not optimized to assess motoric imitation effects and we present performance data here only for completeness (see Table 1, main text, for descriptive data for all three experiments).

**Experiment 1.**

Initiation times. No effect reached significance, main effect of movement compatibility, *F*(1, 29) = 2.28 *p* = .141, *η_p_^2^*= .073, all other *F´s* <1.

Movement times. No effect reached significance, main effect of action-effect compatibility, *F*(1, 29) = 1.01 *p* = .324, *η_p_^2^*= .034, all other *F´s* <1.

Error rates. No effect reached significance, all *F´s* <1.

**Experiment 2.**

Initiation times. No effect reached significance, all *F´s* <1.

Movement times. No effect reached significance, main effect of action-effect compatibility, *F*(1, 29) = 1.15 *p* = .272, *η_p_^2^*= .041, main effect of movement compatibility, *F*(1, 29) = 1.48 *p* = .233, *η_p_^2^*= .049,interaction, *F* <1.

Error rates. No effect reached significance, all *F´s* <1.

**Experiment 3.**

Initiation times. The difference between the action-effect compatible and action-effect incompatible condition was not significance, *t(*54) = 1.31, *p* = .19.

Error rates. The difference between the action-effect compatible and action-effect incompatible condition was significant, *t(*54) = -2.62, *p* = .01.
